# Supplementary material for: Attosecond electron–spin dynamics in Xe 4d photoionization
Source: Nat Commun. 2020 Oct 7;11:5042. doi: 10.1038/s41467-020-18847-1 (PMC7541461; doi:10.1038/s41467-020-18847-1)
Supplement: Supplementary file 1 — Supplementary Information [file 41467_2020_18847_MOESM1_ESM.pdf]

Supplemental Material: Attosecond electron–spin dynamics in Xe 4d photoionization

Shiyang Zhong,[1,*] Jimmy Vinbladh,[2] David Busto,[1] Richard J. Squibb,[3] Marcus Isinger,[1] Lana Neoričić,[1] Hugo Laurell,[1] Robin Weissenbilder,[1] Cord L. Arnold,[1] Raimund Feifel,[3] Jan Marcus Dahlström,[1] Göran Wendin,[4] Mathieu Gisselbrecht,[1] Eva Lindroth,[2] and Anne L'Huillier[1]

[1]*Department of Physics, Lund University, P.O. Box 118, SE-221 00 Lund, Sweden*

[2]*Department of Physics, Stockholm University, AlbaNova University Center, SE-106 91 Stockholm, Sweden*

[3]*Department of Physics, University of Gothenburg, Origovägen 6B, SE-412 96 Gothenburg, Sweden*

[4]*Department of Microtechnology and Nanoscience—MC2, Chalmers University of Technology, SE-412 96 Gothenburg, Sweden*

I. ONE- AND TWO-PHOTON IONIZATION TIME DELAYS

In the main article, we state that the atomic time delays measured through the RABBIT technique reflect the dynamics of one-photon ionization. In this section, we explain the relation between the atomic delays as measured in the experiment to the photoionization time delays shown in Fig. 4. We select the $4d_{3/2} \to \epsilon f_{5/2}$ channel to describe this relationship. Fig. 1 shows the time delay already presented in Fig. 4(c) of the main manuscript (blue line). First, we added back the effect of the Coulomb phase, as shown by the red line. The delay obtained through the RABBIT technique for a single intermediate channel can be expressed with good approximation as a sum of two contributions: $\tau_{\mathrm{A}} = \tau_1 + \tau_{\mathrm{cc}}$ [1, 2], where the first term is the delay discussed previously and the second term, τ_{cc}, shown as a black line, is a correction term, due to IR laser induced continuum-continuum transitions, which is channel-independent over a large energy range [3]. The atomic delay τ_{A} is here calculated in a relativistic framework. In the considered energy range, the difference compared to the Wigner delay (also calculated relativistically) is close to the τ_{cc} obtained non-relativistically using an analytical, universal, i.e. atom and channel-independent formula [2]. Generally speaking, relativistic effects are significant in a rather narrow spectral region close to the 4d threshold, and affect mainly the first (XUV) photon absorption. Fig. 1(a) presents the variation of τ_{A} (green line). As can be observed, the Coulomb phase and the τ_{cc} correction largely compensate each other.

In the RABBIT technique, the atomic delay is extracted by the phase difference divided by 2ω ($2\hbar\omega = 3.1$ eV in our experiment) instead of the actual derivative. The fast oscillations are flattened in the finite difference derivative, as shown by the magenta line in Fig. 1(b). Finally, we compare this result with complete angular-integrated multichannel calculations shown by the black line, which is identical to that in Fig 3 (a). The good agreement between the magenta and black curves implies that the angular integrated atomic delay is dominated by the $4d_{3/2} \to \epsilon f_{5/2}$ channel. Similar agreement is obtained for the $4d_{5/2} \to \epsilon f_{7/2}$ channel. Therefore neglecting the influence of the $d \to p$ channels for simplicity as we did in the manuscript is reasonable. In addition, we demonstrate that the dynamics is governed by one-photon ionization.

* shiyang.zhong@fysik.lth.se

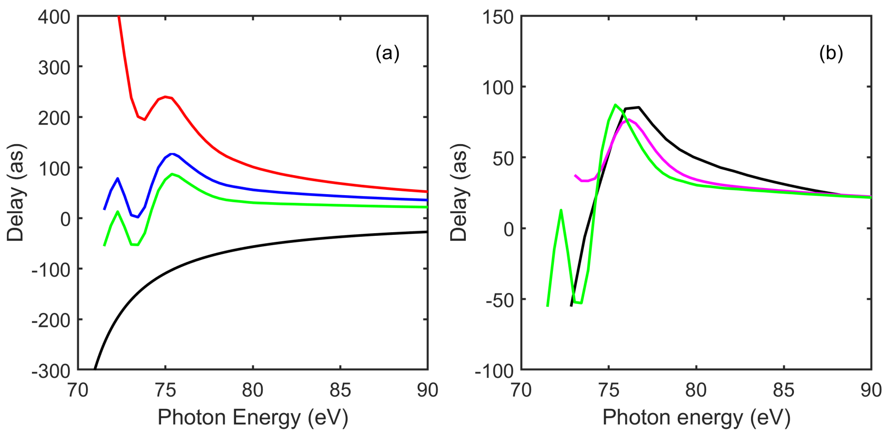

FIG. 1. **Comparison between one- and two- photon calculation.**(a) Wigner delay with (red) and without (blue) the influence of the Coulomb phase for $4d_{3/2} \rightarrow \epsilon f_{5/2}$. The universal $\tau_{\rm cc}$ [2] is shown as a black line. The atomic delay for $4d_{3/2} \rightarrow \epsilon f_{5/2}$ (red+black) is shown by the green line. (b) Angular-integrated atomic delay for $4d_{3/2}$ (black, including all channels), same as in Fig. 3(a) in the manuscript. It is compared with the single channel atomic delay with (magenta) and without (green) the finite difference approximation.

II. MEASUREMENTS OF ABSOLUTE TIME DELAYS

Fig. 3 (a,b) shows differences between Xe $4d$ and Ne $2p$ atomic time delays. A Ne RABBIT scan is taken consecutively after each Xe scan with the same experimental parameters except the gas species, allowing us to remove the influence of the attosecond pulses in this measurement. Fig. 2 (a) presents the atomic time delays for the Ne $2p$ shell in the range of 70 eV to 100 eV photon energy [4]. Because this energy region is well above the $2p$ threshold (21.56 eV), with no sharp spectral features like autoionization or Cooper minimum, $\tau_{\rm A}[{\rm Ne}(2p)]$ remains small, less than ten attoseconds, and can be neglected with respect to the Xe $4d$ time delays. Fig. 2 (b) shows $\tau_{\rm A}[{\rm Xe}(4d_{5/2})]$ (solid line) and $\tau_{\rm A}[{\rm Xe}(4d_{5/2})]$-$\tau_{\rm A}[{\rm Ne}(2p)]$ (dashed line), in addition to the experimental results (dots). The difference between the two theoretical results is negligible, which justifies our approximation.

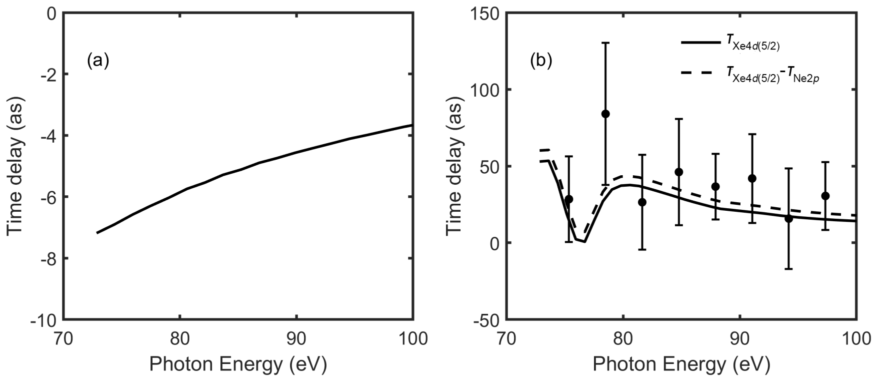

FIG. 2. **Neon 2p shell time delay.** (a) Calculated Ne $2p$ atomic delays in ionization. (b) Calculated (dashed line) and experimental (dot) $\tau_{\rm A}[{\rm Xe}(4d_{5/2})]$-$\tau_{\rm A}[{\rm Ne}(2p)]$ and calculated $\tau_{\rm A}[{\rm Xe}(4d_{5/2})]$ (solid line) using the RRPA.

III. INFLUENCE OF THE $4d \rightarrow \epsilon p$ TRANSITIONS

In the main article, we concentrate on the $4d \rightarrow \epsilon f$ transitions which dominate over $4d \rightarrow \epsilon p$ in the vicinity of the giant dipole resonance. Fig. 3 compares the modulus of the photoionization dipole matrix element and time delay for all transitions. Indeed, the transition strengths for the $4d \rightarrow \epsilon p$ channels are generally much weaker than for $4d \rightarrow \epsilon f$. The $4d \rightarrow \epsilon p$ time delays vary weakly with energy and remain small and negative. They are dominated by the IR correction $\tau_{\rm cc}$ (see below), and converge towards 0 at high energy, similarly to Ne $2p$. In contrast, the $4d \rightarrow \epsilon f$ time delays, which can be interpreted as the time that the f electron spends in the potential barrier of the shape resonance, are much longer. The delay variation observed at low energies present oscillations which probably indicate the influence of spin-flip transitions as for the $d \rightarrow f$ channels, but these are much weaker.

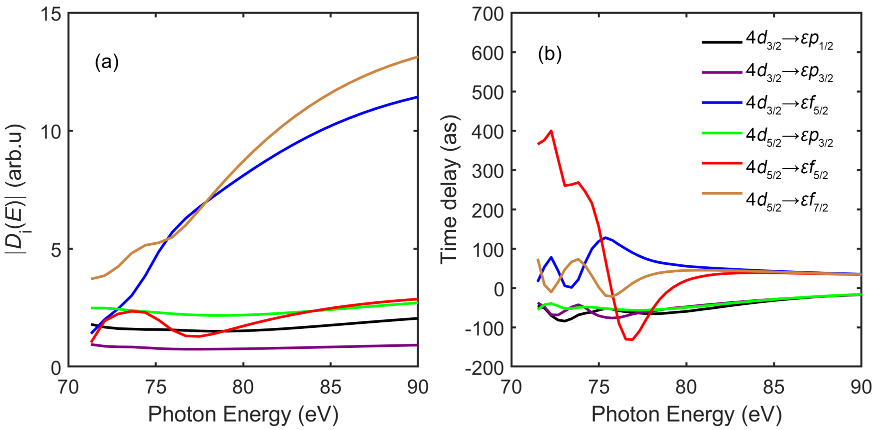

FIG. 3. **Transition matrix elements.** (a) Modulus and (b) time delay for the following transitions: $4d_{3/2} \to \epsilon p_{1/2}$ (black), $4d_{3/2} \to \epsilon p_{3/2}$ (purple), $4d_{3/2} \to \epsilon f_{5/2}$ (blue), $4d_{3/2} \to \epsilon p_{3/2}$ (green), $4d_{5/2} \to \epsilon f_{5/2}$ (red), $4d_{5/2} \to \epsilon f_{7/2}$ (brown). We removed the Coulomb phase-shift in (b).

IV. WIGNER REPRESENTATION

In Fig. 4, we show Wigner representations for the three channels discussed in this article. They exhibit similar features, (i) a broad resonance with a maximum around 100 eV and a short decay; (ii) a sharp resonance at low energy, around 75 eV, with a long decay; (iii) interferences between these resonances. Since the relativistic $4d_{5/2} \to \epsilon f_{5/2}$ channel contributes only weakly to the giant dipole resonance at photon energies larger than 80 eV, the relative amplitude of the narrow feature at 75 eV is much stronger than for the other two channels. We chose to present this result in the main article in order to emphasize the dynamics of relativistic threshold effects.

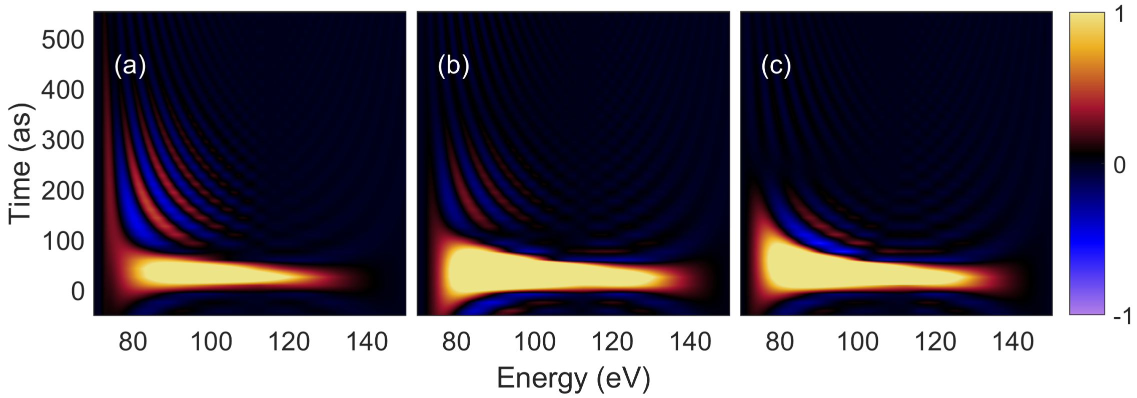

FIG. 4. **Wigner representation.** The wigner representation $W(E,t)$ for (a) $4d_{5/2} \to \epsilon f_{5/2}$ (b) $4d_{5/2} \to \epsilon f_{7/2}$ and (c) $4d_{3/2} \to \epsilon f_{5/2}$ channels. The amplitude is indicated by the color code on the right hand side.

[1] Dahlström, J. M., L'Huillier, A. & Maquet, A. Introduction to attosecond delays in photoionization. J. Phys. B: At. Mol. Phys. **45**, 183001 (2012).

[2] Dahlström, J. M. et al. Theory of attosecond delays in laser-assisted photoionization. Chem. Phys. **414**, 53-64 (2013).

[3] Dahlström, J. M., Carette, T. & Lindroth, E. Diagrammatic approach to attosecond delays in photoionization. Phys. Rev. A **86**, 061402 (2012).

[4] Isinger, M. et al. Photoionization in the time and frequency domain. Science **358**, 893-896 (2017).
